# Metabolism of Gallic Acid and Its Distributions in Tea (*Camellia sinensis*) Plants at the Tissue and Subcellular Levels

**DOI:** 10.3390/ijms21165684

**Published:** 2020-08-08

**Authors:** Xiaochen Zhou, Lanting Zeng, Yingjuan Chen, Xuewen Wang, Yinyin Liao, Yangyang Xiao, Xiumin Fu, Ziyin Yang

**Affiliations:** 1 Key Laboratory of South China Agricultural Plant Molecular Analysis and Genetic Improvement & Guangdong Provincial Key Laboratory of Applied Botany, South China Botanical Garden, Chinese Academy of Sciences, No. 723 Xingke Road, Tianhe District, Guangzhou 510650, China; zhouxiaochen@scbg.ac.cn (X.Z.); zenglanting@scbg.ac.cn (L.Z.); wangxuewen@scbg.ac.cn (X.W.); honey_yyliao@scbg.ac.cn (Y.L.); xiaoyangyang17@scbg.ac.cn (Y.X.); fuxiumin@scbg.ac.cn (X.F.); 2 College of Life Sciences, University of Chinese Academy of Sciences, No. 19A Yuquan Road, Shijingshan District, Beijing 100049, China; 3 Center of Economic Botany, Core Botanical Gardens, Chinese Academy of Sciences, No. 723 Xingke Road, Tianhe District, Guangzhou 510650, China; 4 Department of Tea Science, College of Food Science, Southwest University, No. 2 Tiansheng Road, Beibei District, Chongqing 400715, China; cyj8@swu.edu.cn

**Keywords:** tea, gallic acid, methyl gallate, spatial distribution, subcellular location

## Abstract

In tea (*Camellia sinensis*) plants, polyphenols are the representative metabolites and play important roles during their growth. Among tea polyphenols, catechins are extensively studied, while very little attention has been paid to other polyphenols such as gallic acid (GA) that occur in tea leaves with relatively high content. In this study, GA was able to be transformed into methyl gallate (MG), suggesting that GA is not only a precursor of catechins, but also can be transformed into other metabolites in tea plants. GA content in tea leaves was higher than MG content—regardless of the cultivar, plucking month or leaf position. These two metabolites occurred with higher amounts in tender leaves. Using nonaqueous fractionation techniques, it was found that GA and MG were abundantly accumulated in peroxisome. In addition, GA and MG were found to have strong antifungal activity against two main tea plant diseases, *Colletotrichum camelliae* and *Pseudopestalotiopsis camelliae-sinensis*. The information will advance our understanding on formation and biologic functions of polyphenols in tea plants and also provide a good reference for studying in vivo occurrence of specialized metabolites in economic plants.

## 1. Introduction

Tea is a beverage made from the tender leaves of tea plant (*Camellia sinensis* (L.) O. Kuntze) and has attained considerable popularity owing to its health benefits and good quality [1]. These tea characteristics result from the occurrence of tea metabolites, such as polyphenol, l-theanine, caffeine and aroma compounds [1,2,3]. Therefore, many researchers have focused on the relationships of tea metabolites with tea quality and function [1,4]. Among these metabolites, the content of polyphenols is relatively high, accounting for 18%–36% of tea (dry weight), so they have been studied in-depth [4]. Numerous studies have investigated their contribution to tea quality and human health and metabolism and biologic functions in tea plants. In polyphenols, main focus is on the study of catechin, including its biosynthesis in tea plants and contribution to tea quality [4]. However, there has been relatively few research on gallic acid (GA), another important polyphenol composition. The content of GA is as high as 1% in tea (dry weight) [5], and GA acts as an important contributor to tea taste [6]. Most of current studies have focused on its distribution in different tea products, such as green tea and black tea. The purpose of these studies is to uncover the contribution of GA to tea quality. However, little attention has been paid to the distribution and metabolism of GA in leaves during tea plant growth to explore its potential biologic function. In generally, for human beings, many secondary metabolites act as key quality-related compositions; for plants, they play important biologic functions. Therefore, study on GA distribution in leaves during tea plant growth may help to reveal its potential biologic functions. In addition, at present, it is clear that GA is a crucial precursor of galloylated catechins, the major and characteristic metabolites in tea. It has been revealed that GA can be converted into β-glucogallin, which acts as galloylated acyl donor in the biosynthesis of galloylated catechins [7]. Therefore, GA is considered as the skeleton structure of galloylated catechins. However, it is not clear that whether GA can be converted into other derivatives in tea plants.

As tea genetic transformation system has not been established successfully, in our study, a stable isotope labeling precursor tracing technique was employed to investigate GA metabolism and its derivatives in vivo in tea plants. Moreover, the occurrences of GA and its derivative at the individual plant level, tissue level and subcellular level were investigated, using mass spectrometry and nonaqueous fractionation (NAF) techniques. According to their occurrences and distributions, we also evaluated if GA and its derivative have direct antifungal activity in vitro against the main diseases of tea plants, namely, *Colletotrichum camelliae* and *Pseudopestalotiopsis camelliae-sinensis*. The information will advance our understanding on formation and biologic functions of polyphenols in tea plants and also provide a good reference for studying in vivo occurrence of specialized metabolites in economic plants.

## 2. Results and Discussion

### 2.1. Transformation of GA into MG in Tea Leaves

**Figure 1:**
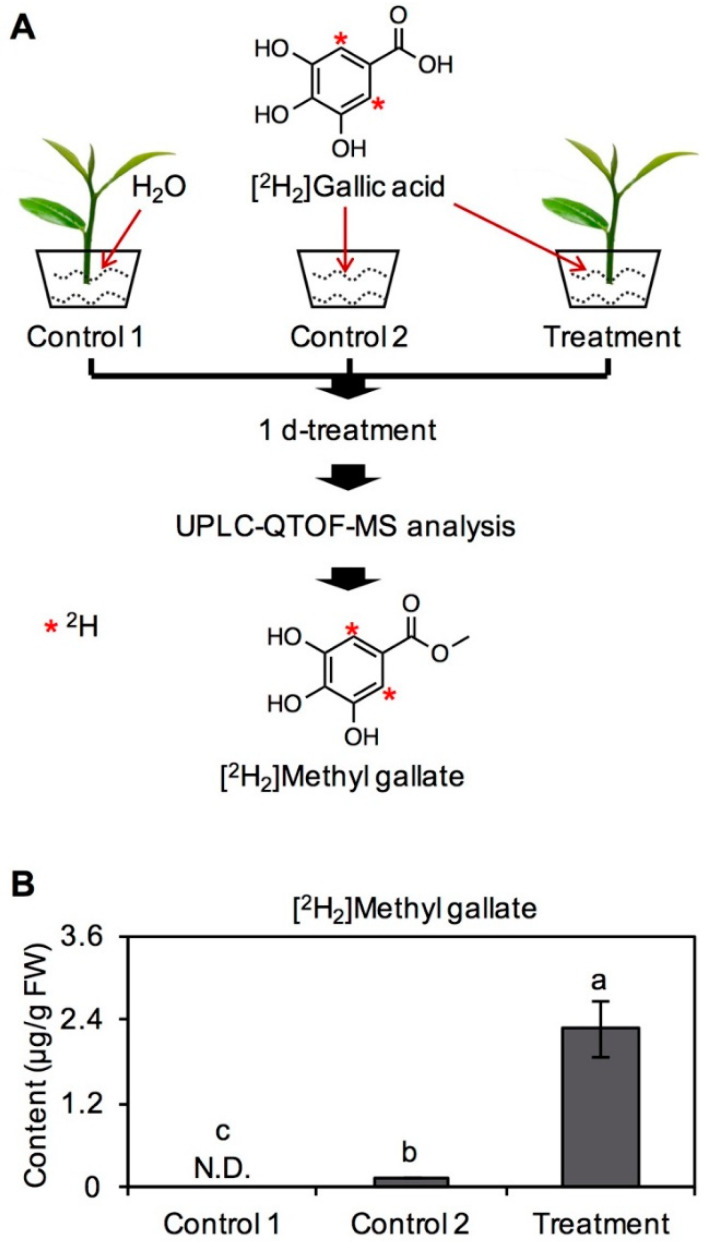
Identification on the transformation of gallic acid into methyl gallate in tea plants. Control 1—tea branches fed with H_2_O; Control 2—only [^2^H_2_]gallic acid solution; Treatment—tea branches fed with [^2^H_2_]gallic acid. As [^2^H_2_]MG may be a byproduct of the chemical synthesis of [^2^H_2_]GA standard, the same amount of [^2^H_2_]GA was used as a control (Control 2). (**A**) Experimental setup of supplement of [^2^H_2_]gallic acid into tea branches. The symbol (*) represents deuterium labelled. (**B**) quantitative analysis of synthesized [^2^H_2_]methyl gallate in tea leaves fed with [^2^H_2_]gallic acid. Data expressed as mean ± standard deviation (SD) (*n* = 3). Means distinguished with different letters are significantly different from each other among different groups.

In the study, commercially purchased [^2^H_2_]GA standard was used to feed tea branches to investigate GA metabolism and its derivatives in vivo in tea plants. After feeding for 1 day, labeled derivatives in tea leaves was identified and quantified by ultra-performance liquid chromatography–quadrupole time-of-flight–mass spectrometry (UPLC–QTOF–MS) (Figure 1A). During the feeding experiment, physiological state of tea leaves was in a good condition because of continuous water supply. Therefore, although the abraded tea branches may be subjected to wounding stress, it makes few effect on the identification of labeled derivatives from [^2^H_2_]GA. [^2^H_2_]MG was obviously detected in fed tea leaves, and none of [^2^H_2_]MG was found in tea leaves without feeding of [^2^H_2_]GA (Figure 1B). As [^2^H_2_]MG is a byproduct of chemical synthesis of [^2^H_2_]GA standard, same amount of [^2^H_2_]GA was used as a control (named as Control 2), and content of [^2^H_2_]MG in [^2^H_2_]GA solution was also determined. After reducing background interference from [^2^H_2_]GA standard, [^2^H_2_]MG was clearly shown to be transformed from [^2^H_2_]GA in tea leaves (Figure 1B). The result showed that GA can act as the precursor of MG, except for galloylated catechins, in tea plants [7].

In vivo tracking through stable isotope-labeled precursor secondary metabolites can help to provide direct evidence of secondary metabolite synthesis [8]. Indeed, stable isotope tracing has been widely applied as an efficacious method to elucidate plant metabolic pathways [9,10]. Furthermore, our previous studies have demonstrated that this method can also be successfully used in tea plants [11,12]. In the present study, using a stable isotope labeling experiment, we confirmed that GA can be transformed into MG in tea leaves (Figure 1). However, the enzymes involved in this pathway have yet to be identified and require further research to obtain more information on biosynthesis pathway of MG from GA in tea plants.

Based on chemical structure, methyl esterase was initially speculated to be involved in the transformation of GA into MG. Therefore, two genes known to encode methyl esterase were selected based on sequence alignment through BLAST. One gene was involved in the transformation of jasmonic acid into methyl jasmonate, while the other gene was involved in the transformation of salicylic acid into methyl salicylate, which has been functionally characterized [13]. In the present study, these two genes were also cloned, expressed using an *Escherichia coli* system and functionally characterized in vitro. However, both recombinant proteins were unable to convert GA into MG in vitro (data not shown). This was perhaps due to the two proteins not being enzymes involved in this methyl esterification process or because the transformation of GA into MG is not converted by methyl esterase. Previous studies have shown that GA can be first glycosylated to 1-*O*-galloyl-β-d-glucose, which then acts as the precursor of galloylated flavan-3-ols [7]. Based on results of in vitro methyl esterase reaction and previous study, it was speculated that more than one step reaction was involved in the conversion of GA into MG. To date, less attention has been paid to the transformation of GA into other forms, such as its methanol conjugate, MG. Therefore, crucial enzymes related to the pathway from GA to MG are of great significance and require further study.

### 2.2. Occurrences of GA and MG in Tea Plants

**Figure 2:**
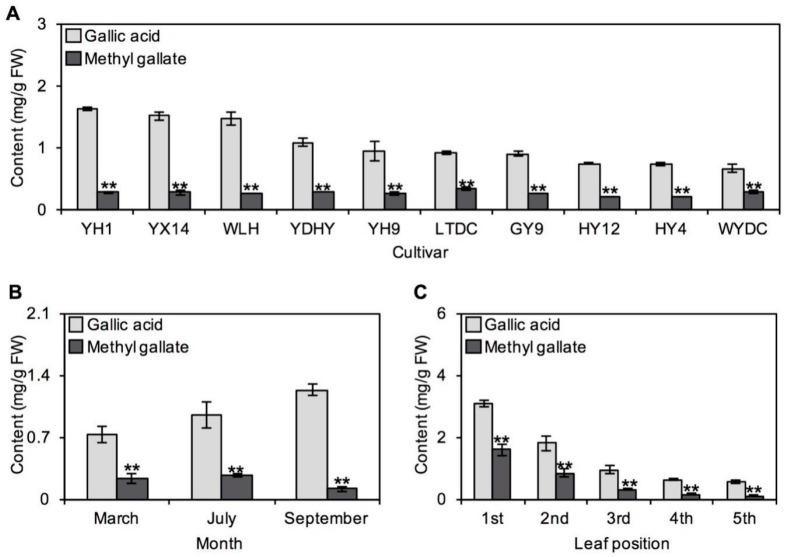
Analysis of gallic acid and its derivative methyl gallate content in tea leaves from different samples. (**A**) Different cultivars. YH1—Yinghong No. 1; YX14—Youxuan No. 14; WLH—Wulinghong; YDHY—Yundaheiye; YH9—Yinghong No. 9; LTDC—Lingtoudancong; GY9—Guanyin No. 9; HY12—Hongyan No. 12; HY4—Hongyan No. 4; WYDC—Wuyedancong; (**B**) different months; (**C**) different leaf position; (**B**,**C**) The tea leaves were from *Camellia sinensis* cv. Yinghong No. 9 plant. Data expressed as mean ± standard deviation (SD) (*n* = 3). Significant differences between gallic acid and methyl gallate content are indicated ** *p* ≤ 0.01). FW, fresh weight.

**Figure 3:**
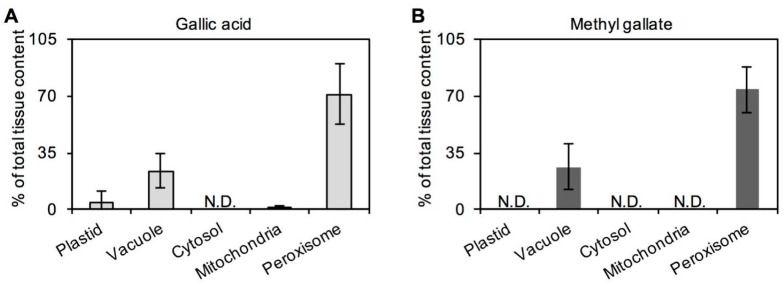
Subcellular distributions of (**A**) gallic acid and its (**B**) derivative methyl gallate in tea leaf tissue. Tea leaves were from *Camellia sinensis* cv. Jinxuan plant. The tissues of tea leaf were fractionated using a nonaqueous procedure. Gallic acid and methyl gallate in each fraction were measured by ultrahigh-performance liquid chromatography. The subcellular distributions were calculated by comparing the metabolite and marker enzyme distributions using Bestfit software. Data expressed as mean ± standard deviation (SD) (*n* = 2). N.D.—not detected.

Based on the results from ten tea cultivars without any treatment, it was found that GA content in tea was always higher than MG content, with the contents of these two metabolites showing 2- to 6-fold differences (Figure 2A). The same relationship was also observed in tea leaves plucked in different months or from different leaf positions (Figure 2B,C). Therefore, GA was concluded to always be present at a higher level than MG in tea leaves, regardless of different tea cultivars, plucking months and leaf positions. Furthermore, tender leaves were found to have higher levels of GA and MG than old leaves (Figure 2C). Correlation analysis of GA and MG contents in tea leaves was also performed. Although MG content increased with increasing GA content, no significant positive correlation was observed between MG and GA contents in tea leaves obtained from different cultivars or plucking months. However, an obvious positive correlation was observed between MG and GA contents in tea leaves obtained from different leaf positions (R^2^ = 0.9996, *p* < 0.0001). In addition, the distributions of GA and MG in different organelles were also analyzed by NAF combined with ultra high-performance liquid chromatography (UHPLC). The results showed that GA and MG were both abundantly accumulated in peroxisomes and vacuoles (Figure 3), exhibiting the same subcellular sites.

Numerous studies have shown that the formation of tea secondary metabolites can be heavily influenced by cultivar, climate and harvest season. Based on metabolic analysis of various tea cultivars, different metabolism was found in various tea cultivars [14,15]. Indeed, GA and MG contents varied across ten cultivars, which may be due to complex genetic background among cultivars (Figure 2A). Environmental factors such as temperature, relative humidity, and light intensity can greatly influence secondary metabolites in plants. Recently, metabolomics has further confirmed that the same phenomena also occurs in tea plants [16,17]. For example, among main aroma compounds responsible for tea quality, the contents of terpene volatiles, such as monoterpenes and sesquiterpenes, change significantly with growing season [18]. In different seasons, different metabolites accumulate in plants with the potential aim of adapting to variational environments. It was found that biosynthesis and signal transduction of catechins are closely regulated by various abiotic and biotic stresses [15], suggesting that catechins may play a key role in tea plant–environment interactions. In this study, GA and MG contents were different in different harvest months, which may be a response to environment (Figure 2B). Furthermore, there are significant differences in GA content between these harvesting months. However, based on the present results, it was not enough to draw a conclusion that older plants tend to produce higher levels of GA. Because the tea leaves plucked in different months were all first three leaves from nearly same aged tea plants. In the present materials, the variable environmental conditions in different months may have the stronger effect on the level of GA than the age of tea plants. The determination of GA content in same-position leaves from tea plants with different ages may help to answer the question. In addition, changes in leaf position also caused a difference in tea metabolites, accounting for material selection of tender leaves (one bud and two or three leaves) in tea production. In this study, GA and MG contents were higher in tender leaves from *C. sinensis* Yinghong No. 9 (Figure 2C). This phenomenon may be ubiquitous in tea plants, as the same tendency was also observed in another tea cultivar [18].

Owing to the lack of a genetic transformation system for tea plants, in vivo evidence of metabolite biosynthesis locations is difficult to obtain. Direct investigations on the localization of tea metabolites at the cellular and subcellular levels may help to solve this problem to some extent. NAF method has mostly been applied in plants to determine general metabolites in leaves of *Arabidopsis* and spinach [19] and potato tube [20]. Plant cell is highly compartmentalized with multiple organelles surrounded by membranes that perform its distinct functions to maintain cell physiology. NAF first, established in 1978, has been continuously optimized to recalculate subcellular metabolite distributions among organisms. The principle about NAF method is shown as follows [21]: during the extract procedure (for example grinding, freeze-drying and ultrasonication), particles that contain larger or smaller parts of a respective subcellular compartment were generated. These particles contain enzymes, membranes, metabolites, etc. and are stable in heptane/tetrachlorethylene. Based on the enrichment of compartments within a continuous nonaqueous density gradient and the correlation of enzyme activity of organism-specific marker with metabolite content, the subcellular distributions of each compound in the NAF fractions can be calculated. Recently, the method has been successfully used to investigate the subcellular distribution of characteristic metabolite in tea plants [22]. In this study, we had reconfirmed that this method was feasible in tea leaves based on density optimization. Obvious positive correlation between GA and MG based on NAF combined with UHPLC analysis demonstrated that GA may be transformed into MG in tea leaves (Figure 3). Furthermore, subcellular analyses of GA and MG showed that they are mostly accumulated in peroxisomes, suggesting that the enzymes involved in the transformation of GA into MG may be located in peroxisomes. Although methylation modification reactions mainly occur in the cytosol, a previous study has proposed that some may take place in other subcellular compartments, such as peroxisomes [23]. However, more evidence is needed to confirm this speculation.

### 2.3. Antifungal Properties In Vitro of GA and MG against Main Tea Pathogens

**Figure 4:**
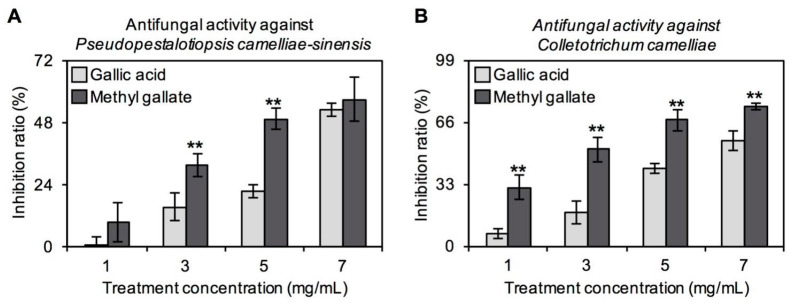
Evaluation of antifungal ability in vitro of gallic acid and its derivative methyl gallate. Comparison analysis of antifungal function in vitro between gallic acid and methyl gallate against (**A**) *Pseudopestalotiopsis camelliae-sinensis* and (**B**) *Colletotrichum camelliae*, two main pathogens of tea plants. Data expressed as mean ± standard deviation (SD) (*n* = 4). Significant differences between two groups are indicated (** *p* ≤ 0.01).

Some studies have reported that GA and its derivate MG have antimicrobial effects in vitro and in vivo [24,25,26,27]. However, in these investigations, none of the pathogens used for resistance evaluation were the main pathogens of tea plants. The information was unable to support the antimicrobial function of GA and MG in tea plants. Therefore, in the study, two main pathogens in tea plants, *Pseudopestalotiopsis camelliae-sinensis* and *Colletotrichum camelliae*, were used to investigate the antifungal properties of GA and MG in vitro. With increasing concentration of these two pathogens, the antifungal abilities of GA and MG both increased (Figure 4). To *Colletotrichum camelliae*, MG was found to have greater antifungal activity than GA at any treatment concentration. There were also significant differences between GA and MG on inhibition ratio of *Pseudopestalotiopsis camelliae-sinensis* when the treatment concentrations were 3 mg/mL and 5 mg/mL. However, when the treatment concentrations were 1 mg/mL and 7 mg/mL, there was no significant difference (Figure 4). This activity was most obvious against *Pseudopestalotiopsis camelliae-sinensis*, with EC_50_ of 7.43 mg/mL and 5.36 mg/mL for GA and MG, respectively. Furthermore, EC_50_ values against *Colletotrichum camelliae* were 6.47 mg/mL and 2.44 mg/mL for GA and MG, respectively.

According to the organs in which they occur, tea plant diseases are mainly divided into four types, namely, leaf disease, stem disease, root disease and flower disease. As raw materials for tea products are tea leaves and buds, the impact of leaf disease on yield is more important and has attracted more attention. Among these diseases, gray blight disease caused by *Pestalotiopsis theae* and anthracnose caused by species of genus *Colletotrichum* are the most serious diseases and have been reported to cause significant production and quality losses in many commercial tea cultivation areas [28,29]. *Pestalotiopsis theae* can invade various leaves, including tender, mature and old tea leaves, causing large brown–black spots at the latter stages of the disease and forming apparent concentric rings [30]. Therefore, initial brown–black spots may be an early symptom of gray leaf blight disease. *Colletotrichum camelliae* is the dominant *Colletotrichum* species on Chinese tea plants and is probably host-specific to Camellia [31]. As tea leaves have a thick cuticle, *Colletotrichum* does not easily infect non-wounded leaves, but is highly pathogenic to wounded leaves [32]. To date, these diseases are difficult to control, because their molecular mechanisms remain poorly understood. Host plants may have evolved multiple defense mechanisms in their interactions with various pathogens. Many studies have attempted to elucidate the molecular mechanisms of tea plant resistance against pathogens [33,34]. Additionally, plants can produce many chemicals, known as secondary metabolites, to protect themselves.

In tea plants, GA and MG may be candidates for resistance against diseases induced by *Pseudopestalotiopsis camelliae-sinensis* and *Colletotrichum camelliae* (Figure 4). Compared with older leaves, tender leaves have a thinner waxy layer, resulting in weaker physical defense. Therefore, high contents of GA and MG, which have strong antifungal abilities, in tender tea leaves may be conducive to their chemical defense during growth (Figure 2C). However, further studies are required to investigate the inference. It was found that GA affects the physiological growth of filamentous fungi [35]. Similarly, some studies have found that MG has strong oxidative and antimicrobial abilities. The antimicrobial properties of MG against microorganisms, such as *Shigella dysenteriae*, *Escherichia coli* and *Salmonella*, have been reported [26,27]. The purpose of this study was to confirm the antifungal activity of MG and GA against the two main tea plant diseases, providing the preliminary information on potential biologic functions of MG and GA during tea plant growth. The underlying mechanism is of interest and needs in-depth exploration. Usually, the antimicrobial action modes are diverse, including disrupting antimicrobial cell membrane and inhibiting intracellular targets [36]. Previous study showed that MG exhibits antimicrobial activity by destroying the cell membrane integrity, resulting in physiological variations to alter the membrane potential and decreasing cellular ATP levels [37]. In the present study, it remains to be determined whether GA and MG also destroy cell membrane integrity to inhibit *Pseudopestalotiopsis camelliae-sinensis* and *Colletotrichum camelliae*. MDA level is one of key indicators for reflecting the membrane integrity and may be used to investigate the antimicrobial action modes of GA and MG against tea plant-specific pathogens. In addition, a study showed that MG is more efficient than GA to inhibit an oxidative stress in a biologic system [38]. Therefore, the stronger antioxidant of MG compared with that of GA may account for its higher antifungal abilities against *Pseudopestalotiopsis camelliae-sinensis* and *Colletotrichum camelliae* in vitro (Figure 4).

## 3. Materials and Methods

### 3.1. Plant Materials

Tea leaves from *C. sinensis* cv. Yinghong No. 9, Jinxuan, Yundaheiye, Guanyin No. 9, Hongyan No. 4, Hongyan No. 12, Lingtoudancong, Yinghong No. 1, Youxuan No. 14, Wulinghong and Wuyedancong were used to investigate in the study. All tea cultivars were planted at Yingde Tea Experimental Station of the Tea Research Institute, Guangdong Academy of Agricultural Sciences (Yingde, Guangdong, China).

### 3.2. Feeding Experiment in Tea Leaves with [^2^H_2_]GA and Determination of [^2^H_2_]MG

Tea branches from *C. sinensis* cv. Yinghong No. 9 plucked in June 2019 were applied to investigate the transformation of GA into MG in tea leaves. Tea branches with four leaves were cultivated in 6-mM [^2^H_2_]GA solution and kept in controlled conditions (temperature 25 ± 2 °C; humidity 70%; light/dark photoperiod, 16 h/8 h) for 1 d. Tea branches cultivated in H_2_O under the same conditions were used as control. During the feeding treatment, the tea leaves were maintained in a good physiological state because of continuous supply of water. As there is [^2^H_2_]MG in the [^2^H_2_]GA standard, only 6-mM [^2^H_2_]GA solution kept under the same conditions was set as another control. Four replicates were kept for each treatment. After treatment, samples were frozen with liquid N_2_ and kept at −80 °C for future study.

Finely powdered sample (200 mg, fresh weight) was extracted with 2 mL methanol by ultrasonic treatment in ice-cold water for 20 min. Extracts were filtered through a 0.22 μm membrane and subjected to an UPLC–QTOF–MS (Acquity UPLC I-class/Xevo^®^ G2-XS QTOF, Waters Corporation, Milford, MA, USA) [11]. Each sample (2 μL) was injected into a Waters ACQUITY UPLC HSS T3 C18 column (2.1 mm × 100 mm i.d., 1.8 μm). The mobile phase consisted of water with 0.1% (*v/v*) formic acid (A) and acetonitrile with 0.1% (*v/v*) formic acid (B) with an initial condition of 90% of mobile phase A and 10% of mobile phase B. The linear gradient elution was performed as follows: 0–10 min, 10%–100% B; 10–13 min, 100%–100% B; 13–13.1 min, 100%–10% B; 13.1–16 min, 10% B. The flow rate was 0.3 mL/min. The column temperature was 40 °C. Mass spectrometry was recorded using a Xevo G2-XS QTOF equipped with an ESI source and controlled by MassLynx v 4.1 software. A full MS scan was performed in the range *m/z* 50–1000 Da at a sensitivity mode with scan time 0.5 s. The electrospray ionization was operated on negative mode. The capillary voltages were set at 2 kV, and the cone voltage was 40 V. The source temperature was 100 °C, and the desolvation temperature was 250 °C. Nitrogen gas was used both for the nebulizer and in desolvation. The cone gas flow rates and desolvation were 50 L/h and 550 L/h, respectively. The low energy was set as 6 V, and high energy was ramp of 25 eV to 35 eV. The identification of [^2^H_2_]MG was based on the retention time and *m/z* data, i.e., [^2^H_2_]MG (t_R_ = 3.25 min, *m/z* 185.0419 [M-H]^−^, calcd. for C_8_H_6_^2^H_2_O_5_). The quantitative analysis of [^2^H_2_]MG was based on calibration curve, which was constructed by plotting the concentration against the peak area of MG authentic standard.

### 3.3. Extraction and Analysis of GA and MG in Tea Leaves

Tea leaves (first three leaves) from *C. sinensis* cv. Yinghong No. 9, Yundaheiye, Guanyin No. 9, Hongyan No. 4, Hongyan No. 12, Lingtoudancong, Yinghong No. 1, Youxuan No. 14, Wulinghong and Wuyedancong plucked in July 2019 were used to investigate the distribution of GA and MG in tea leaves from different cultivars. Tea leaves (first three leaves) from *C. sinensis* cv. Yinghong No. 9 plucked in March 2018, July 2019 and September 2018 were used to investigate the distribution of GA and MG in tea leaves from different months. First, second, third, fourth and fifth leaf from *C. sinensis* cv. Yinghong No. 9 plucked in September 2019 were used to investigate the distribution of GA and MG in tea leaves from different positions. Three replicates were kept for each sample.

Finely powdered tea leaves (300 mg, fresh weight) were extracted with 2 mL methanol by ultrasonic treatment in ice-cold water for 20 min. Extracts were filtered through a 0.22-μm membrane and analyzed by UHPLC (Ultimate 3000, Thermo Scientific, Waltham, MA, USA) fitted with Hypersil GOLD column (2.1 mm × 100 mm i.d., 1.9 μm). The flow rate was 0.2 mL/min. The column temperature was 40 °C. The mobile phase contained acetonitrile solution (A) and water with 0.2% (*v/v*) formic acid (B) with an initial condition of 95% of mobile phase B. The linear gradient elution was carried out as follows: 0–2.5 min, 95%–89% B; 2.5–10 min, 89% B; 10–13 min, 89%–5% B; 13–16 min, 5% B; 16–16.1 min, 5%–95% B; 16.1–19 min, 95% B. The targeted wavelength was 280 nm for GA and MG. The quantitative analyses of GA and MG were based on calibration curves obtained from the authentic standards.

### 3.4. Determination of Subcellular Distribution of GA and MG

The tea leaves from *C. sinensis* cv. Jinxuan plucked in March 2019 were fractionated using a nonaqueous procedure according to the published studies with a slightly modified [20,21,39]. The finely powered tea leaves (4 g, fresh weight) was placed into the lyophilizer at 0.02 bar and −50 °C for 3 days. The dry powder was resuspended in tetrachlorethylene–heptane mixture (20 mL, 66:34 (*v/v*); density = 1.3 g/cm^3^; the solvents were stored with 3 Å molecular sieve) and ultrasonicated for 2 min with 6 cycles of 10 s pules and 10 s breaks at 65% power. The suspension was filtered through nylon net with a pore size at 20 μm, washed the net 3 times with 10 mL of heptane and centrifuged for 10 min at 3200 *g* and 4 °C. After centrifugation, the organic supernatant was removed and the pellet was resuspended in C_2_C_l4_/C_7_H_16_ mixture (3 mL, 66:34 (*v/v*)). Aliquots (500 μL with 10 × 50 μL) were withdrawn for determination of metabolites and enzyme activity in the unfractionated material, and the remaining 2.5 mL of the suspension was loaded on the top of the gradient. A linear gradient (25 mL, for leaf tissue between 1.43 and 1.50 g/cm^3^) was made using a gradient former connected to a peristaltic pump. The gradients were centrifuged for 1 h at 3800 *g* at 4 °C. The fractions (F1 to F5, 4–6 mL for each fraction) was carefully removed from the top using Pasteur pipettes into a clean 50-mL tube. Three volumes of C_7_H_16_ were added to each tube and the mixture was mixed well. The suspensions were centrifuged for 10 min at 3200 *g* at 4 °C in a swing-out-rotor centrifuge. The supernatants were discarded, and the pellet was resuspended in 5 mL C_7_H_16_ and 10 aliquots of 500 μL of the suspension were transferred into 2-mL tubes. The sample in tube was dried by N_2_ for 1 h and then extracted for assay of metabolites (GA and MG) and enzymes (proteins).

The metabolites (GA and MG) from the dried samples were resolved in 1 mL 80% methanol and subjected to ultrasonic treatment for 10 min. The solutions were filtered through a 0.22-μm membrane and analyzed by UHPLC as above. Proteins from the dried samples were extracted by buffer A (50-mM Hepes–NaOH pH 7.4; 1.5-mM PMSF; 1-g/L PVPP; 1-mM EGTA; 1-mM EDTA; 2-mM aminocaproic acid; 2-mM benzamidine; 5-mM MgCl2; 0.1% Triton X-100; 10% glycerol) for determined the contents of proteins of GAPDH (plastid marker), UGPase (cytoplasm marker), CAT (peroxisome marker) and cytochrome C oxidase (mitochondria marker); proteins from the dried samples were extracted by buffer B (0.5-M sodium acetate pH 5.0 adjusted using glacial acid) for determined the contents of proteins of AP (vacuole marker).

### 3.5. In Vitro Antifungal Experiment

The antifungal activity in vitro of GA and MG was judged by their inhibitory effect on the growth of *Pseudopestalotiopsis camelliae-sinensis* and *Colletotrichum camelliae*, which are the main pathogens of tea plants [40]. The test chemicals were firstly dissolved in DMSO. Each chemical solution was mixed with potato dextrose agar (PDA) culture media to obtain final concentrations of 0, 1, 3, 5 and 7 mg/mL. In the prepared PDA, DMSO used for dissolving chemicals was diluted into very low concentration. In addition, to counterbalance the effect of DMSO, the PDA only with equal volume of DMSO was used as the control. A mycelial disk with 4-mm diameter of the test pathogens obtained from the 5-day-old culture was placed at the center of PDA medium. Each treatment was repeated four times and kept at 25 ± 2 °C for 5−7 days. The percentage inhibition of the growth of *Pseudopestalotiopsis camelliae-sinensis* and *Colletotrichum camelliae* by chemicals, compared with the controls, were calculated at 5 days and 6 days, respectively. Percentage of mycelial inhibition (%) = [(C-T)/C] × 100, where C and T are average colony diameters in control and treatment, respectively. The fungicide concentration that caused 50% mycelium growth inhibition (EC_50_) was calculated from the average of colony diameters. We used the SPSS statistics package to determine EC_50_ value by probit–log analysis (Version 20.0, Chicago, IL, USA).

### 3.6. Statistical Analysis

SPSS Statistics package (Version 23.0, Chicago, IL, USA) was applied to carry out statistical analysis. The differences between two groups and among three or more than three groups were determined by Two-tailed student’s *t*-test and one-way analysis of variance (ANOVA) follow by Duncan’s multiple comparison tests, respectively. A probability level of 5% (*p* ≤ 0.05) was regarded as significant. All data are expressed as the mean ± standard deviation (SD).

## 4. Conclusions

**Figure 5:**
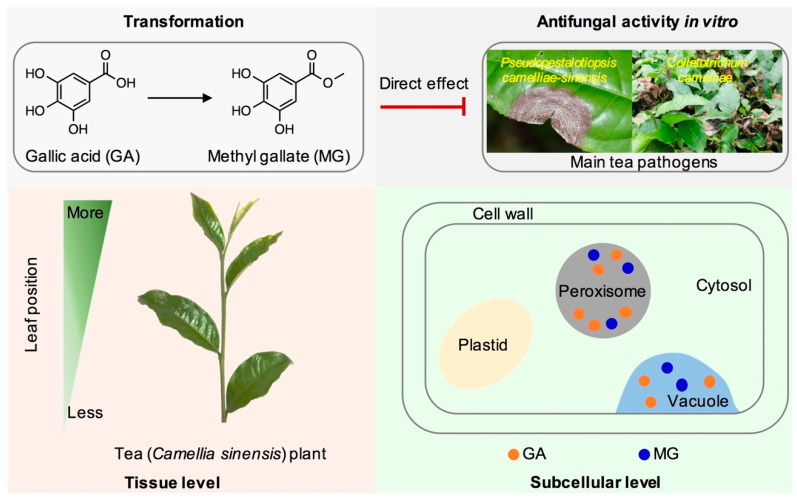
Summary of the occurrence of gallic acid and its derivative methyl gallate in tea leaves. Both gallic acid and methyl gallate have antifungal activity in vitro against *Pseudopestalotiopsis camelliae-sinensis* and *Colletotrichum camelliae*, which are main pathogens in tea plants.

Herein, multidomain approaches, including stable isotope tracing experiments, chromatographic analysis, NAF combined with UHPLC analysis and antimicrobial property analysis in vitro have been used to comprehensively investigate the metabolism, occurrence, and antifungal properties of GA and MG in tea leaves (Figure 5). Stable isotope tracing experiments showed that GA can be transformed into MG in tea leaves. Significantly more GA than its derivate MG is present in tea leaves, regardless of the cultivar, plucking month and growth position. The positive correlation between GA and MG contents in leaves from different positions showed that tender leaves contained higher amounts of both metabolites. Furthermore, the correlation of these two metabolites in tea leaves was further confirmed at the subcellular level. GA and MG had the strong antifungal effects against major tea pathogens *Pseudopestalotiopsis camelliae-sinensis* and *Colletotrichum camelliae*. This study provides information concerning the occurrence and metabolism of important polyphenols in tea plants.

## Abbreviations

FW	Fresh weight
GA	Gallic acid
MG	Methyl gallate
NAF	Nonaqueous fractionation
UPLC–QTOF–MS	Ultra-performance liquid chromatography/quadrupole time-of-flight mass spectrometry
UHPLC	Ultra-high–performance liquid chromatography
